# London Hospital Nurses: Miss Lynette Gould's View; Miss McKinley Withdraws Her Statements

**Published:** 1918-08-24

**Authors:** Helena McKinley


### THE EDITOR'S LETTER-BOX.

(*Continued from page* 451.)

### LONDON HOSPITAL NURSES.

### Miss McKinley Withdraws her Statements.

SIR,—I have had under very careful consideration my letter dated July 27 last, which you published on page 397 of THE HOSPITAL of the 3rd instant, your article thereon published on page 415 of THE HOSPITAL of August 10, and the fact that no evidence is forthcoming from your readers in support of the statements my letter contained. Those statements were made in good faith and rest upon statements made to me by nurses who have passed from under my control into the hospital world, and whose present addresses I do not know. Not one of them has come forward in support of my letter, as I had a right to expect they would do, and in all the circumstances it seems to me that the right course for me to take is to express regret and withdraw my letter and the statements it contains.

I am deeply attached to my profession, a keen member of the College of Nursing, yielding to no one in my desire to uphold its high standard. I recognise the justice of your claim that it is due to my profession, "in the absence of any such complaints, that the London Hospital should no longer suffer amongst trained nurses from floating statements, which have never been demonstrably proved to relate to its conditions of nurse training at the present time." I therefore withdraw my letter, believing in unity in the profession, and justice for all.—I am, Sir, yours faithfully,

(Signed) HELENA MCKINLEY.

